# The fantastic voyage: primordial germ cell migration through the developing mouse embryo

**DOI:** 10.1042/BST20253009

**Published:** 2025-07-17

**Authors:** Katharine Goodwin

**Affiliations:** MRC Laboratory of Molecular Biology, Cambridge Biomedical Campus, Cambridge CB2 0QHU.K

**Keywords:** cell adhesion, cell migration, germ cells, mechanobiology, morphogenesis

## Abstract

During the early stages of embryonic development, a small population of cells is set aside to form the germline. These primordial germ cells (PGCs) are often specified separate in time and space from their eventual home, the developing gonads. PGCs must therefore undertake a journey through the developing tissues of the embryo to reach their destination and ensure the fertility of the organism. Despite decades of interest in this topic and significant progress made in other model organisms, there is still very little known about how this migration is accomplished in the mouse or any other mammal. In this review, I explore over half a century of work examining this enigmatic cell and its voyage through the mouse embryo, interpreting these findings in light of recent discoveries in the field of cell migration. I discuss possible migration modes used by mouse PGCs, changes in their microenvironment that could influence migration, and roles the nucleus might play in their journey. With modern advances in microscopy and transgenic reporter mice, it is time to delve into this fascinating and important model of cell migration *in vivo*.

## Introduction

Animal reproduction relies on the formation of the germline, a process which begins in the early embryo. Germ cells are typically specified before the formation of the gonads that they will eventually colonize. In many species, from *Drosophila* to human, primordial germ cells (PGCs) in the early embryo must therefore travel through developing tissues to arrive at the correct location, the gonadal ridge. PGC migration is an evolutionarily conserved process essential for fertility, but there are differences in the migration strategies and routes among species [1–3]. *Drosophila*, mouse, and human PGCs, for example, all travel with the developing hindgut, while chicken PGCs spend part of their journey in the vasculature [2,4]. Depending on the size and developmental rate of the embryo, as well as the time and place of PGC specification, the duration and distance of PGC migration and the obstacles that PGCs encounter vary widely. PGC migration in different species is therefore a model for studying cell migration *in vivo* through diverse environments.

**Figure 1:**
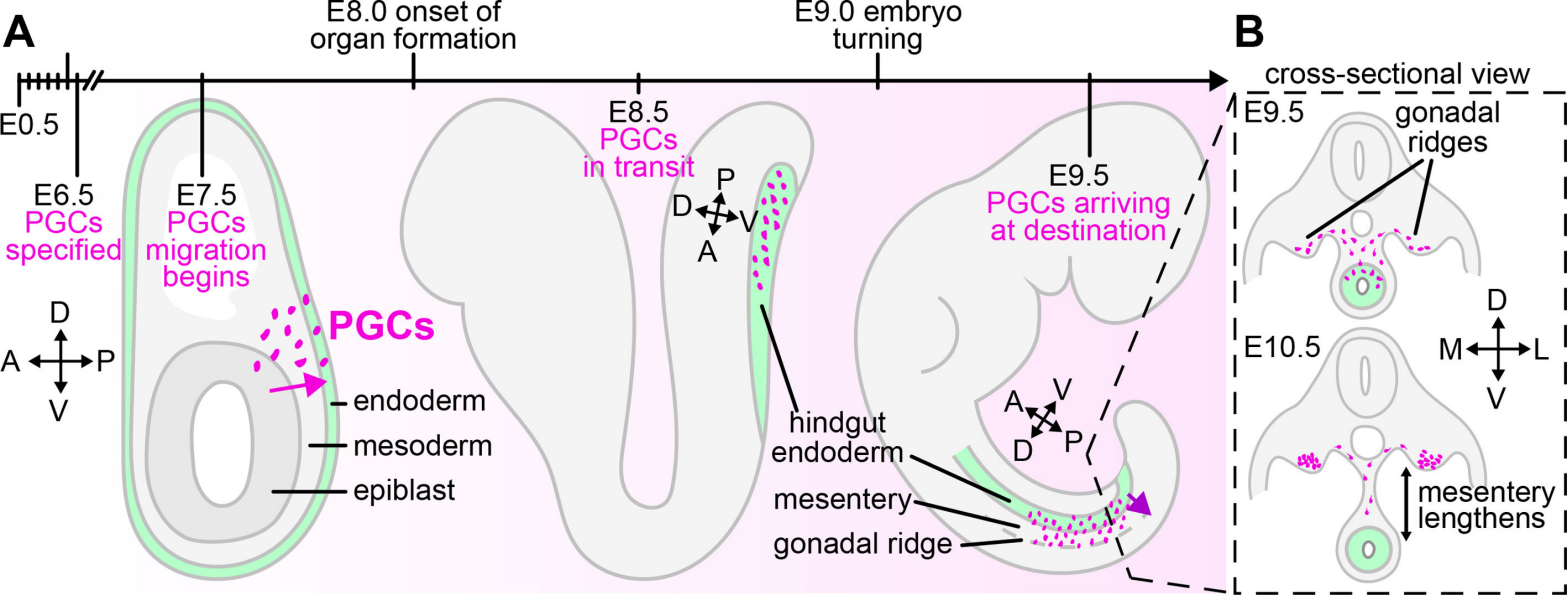
Route of PGC migration through the developing mouse embryo. (**A**) Schematics of E7.5, E8.5, and E9.5 embryos illustrating the major steps in PGC migration and the relevant tissues. Relevant events along this developmental timeline are included, and embryonic axes of the portion of the embryo containing PGCs are indicated. Magenta arrows indicate the path of directed PGC migration. (**B**) Cross-sectional view of E9.5 and E10.5 embryos showing migratory path of PGCs and how this is affected by morphogenesis of the dorsal mesentery. PGCs, primordial germ cells.

**Figure 2:**
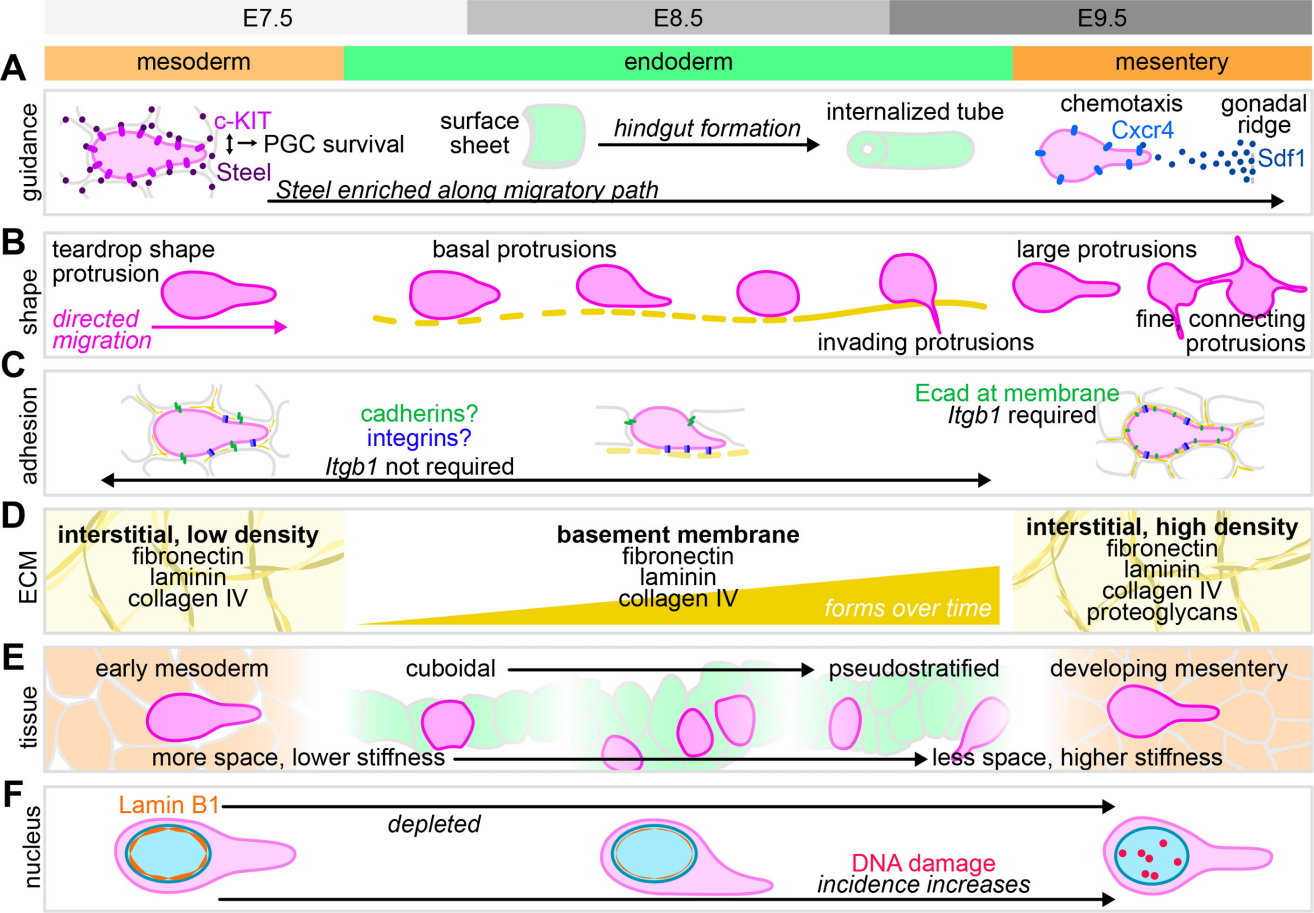
PGC migration modes and microenvironment. (**A**) Guidance cues during PGC migration, including molecular interactions and the formation of the hindgut that internalizes PGCs into the embryo. Commonly used protein names in the PGC field are shown, including c-KIT (gene symbol: *Kit*), Steel (*Kitl*), Cxcr4 (*Cxcr4*), and Sdf1 (*Cxcl12*). (**B**) PGC shapes and protrusions at each developmental stage and in each tissue (mesoderm, endoderm, mesentery). (**C**) Possible adhesions made by PGCs in each tissue. E-cadherin (Ecad) is found at the PGC membrane in the Ecad-negative mesentery. Stages where *Itgb1* is required are indicated.(**D**) ECM environment around migrating PGCs, including interstitial ECM in the mesoderm and mesentery and basement membrane development during PGC residence in the endoderm. (**E**) Tissue architecture around migrating PGCs, including changes to endodermal architecture, intercellular spaces, and tissue stiffness. (**F**) The incidence of DNA damage in PGCs increases as the tissues around them develop. Concomitantly, they deplete Lamin B1. ECM, extracellular matrix; PGCs, primordial germ cells.

In the mouse embryo, PGCs are specified from the somatic cells of the posterior epiblast at embryonic day (E) 6.5. They migrate through the mesoderm to reach the endoderm on the surface of the embryo through E7.5 [5,6] and are then internalized into the embryo as the endoderm invaginates to form the hindgut through E8.5 [7,8] (Figure 1A). At E9.5, they begin a gradual exodus from the hindgut endoderm into the surrounding mesentery and migrate to the gonadal ridges [9] (Figure 1A and B). The signals that guide migration are described in more detail elsewhere [1], and we still lack a complete understanding of the multiple and possibly redundant guidance cues throughout PGC migration in the mouse. Throughout their journey, PGCs express c-KIT and receive survival signals via Steel from the somatic cells specifically on their migration path (Figure 2A) [10–12]. The signals that cause them to home to the endoderm at E7.5 are not fully elucidated, but once they are in the endoderm, the formation of the hindgut is responsible for relocating PGCs for the final leg of their journey (Figure 2A) [7]. This last step is primarily guided by Sdf1, released by the gonadal ridge, and sensed by the Cxcr4 receptor on PGCs (Figure 2A) [13,14].

This multiple-day journey includes migration through both mesodermal and endodermal tissues, characterized by different extracellular matrix (ECM) organization and tissue architecture. Furthermore, the embryo undergoes significant morphogenesis from E7.5 to E9.5, with the establishment of the body plan and the onset of organogenesis. This causes further changes to the tissue organization around migrating PGCs. Cells in culture adapt their migration modes to different microenvironments [15,16], and zebrafish PGCs exhibit different protrusive behaviors in mesodermal and ectodermal tissues [17]. How PGCs in the mouse embryo migrate through diverse environments and adapt to the changing conditions around them is unknown.

The mechanisms of PGC migration have been most extensively studied in *Drosophila* and zebrafish embryos, which are amenable to fast genetic manipulation and live imaging. We know comparatively very little about PGC migration in mammals, despite long-standing interest in the process [18,19], due to the difficulties associated with manipulating and imaging post-implantation mammalian embryos. However, with advancements in imaging and culture techniques and the increasing prevalence of fluorescent reporter mice, this fascinating process is ready to revisit. Here, I assemble evidence from decades of sparse investigations into PGC migration in the mouse and interpret it in the context of modern discoveries in the field of cell migration. The focus is on the mechanisms of migration, including migration modes and the roles of the extracellular environment, morphogenesis of the surrounding tissues, and the nucleus.

### PGC migration mode in mouse

Depending on the cell type and the surrounding microenvironment, cells migrating in 3D employ different migration modes ranging from mesenchymal to amoeboid [20]. Mesenchymal migration is characterized by a long, actin-rich protrusion at the cell front, strong adhesion to the surrounding ECM, and proteolytic activity to degrade the ECM in the migratory path [20]. Amoeboid migration, which encompasses a wide variety of migration modes, is characterized by blebs at the cell front and low adhesion to the substrate [20,21]. Both *Drosophila* and zebrafish PGCs use migration modes closer to amoeboid, but their exact mechanisms of migration differ. *Drosophila* PGCs remain rounded throughout active migration and generate actomyosin contractility-dependent retrograde actin flows along the length of the cell [22]. Zebrafish PGCs have similar global retrograde actin flows [23] but migrate with a large bleb at the cell front that is devoid of actin and bordered by E-cadherin to localize retrograde actin flow to the bleb margins, effectively maintaining cell polarity in the direction of migration [24,25].

The migration mode(s) used by mouse PGCs are not definitively known, but evidence from electron microscopy (EM) studies, fixed samples, and live imaging suggest a different approach to migration. Early EM studies showed that PGCs in the dorsal mesentery make large, irregular cytoplasmic protrusions [18,26] (Figure 2B). They also form long thin protrusions that contact other PGCs, which may be involved in migration or communication [27] (Figure 2B). Live imaging showed that migrating PGCs at E7.5 extend long, thin protrusions and maintain a polarized, teardrop shape [5] (Figure 2B). Through E8.5, when PGCs are in the endoderm, they remain motile and extend protrusions that are confined to the endoderm [5,9] (Figure 2B). At E9.5, these protrusions begin extending into the mesentery [9] (Figure 2B). The long protrusions observed at the front of migrating mouse PGCs are more reminiscent of a mesenchymal migration mode. In line with this, recent live imaging work shows that these protrusions precede PGC migration and have high levels of the actin reporter LifeAct-RFP [28]. Additionally, PGCs express a repertoire of matrix metalloproteinase (MMP) and related genes that enable proteolytic remodeling of their microenvironment [29]. To fully define the migration mode(s) used by PGCs in the mouse embryo, we would need to determine where contractility and retrograde actin flows are generated. This should be possible via live imaging of embryos expressing actin reporters [30] or biosensors providing readouts of actomyosin contractility [31] alongside PGC reporters, such as the Oct4ΔPE-GFP mouse line [32]. Additionally, advances should be made to target expression of reporters to PGCs specifically, using either inducible reporters and Cre lines (e.g., Prdm1-Cre [33] or Nanos3-Cre [34]) or by placing reporters under the control of the truncated Oct4 promoter (Oct4ΔPE) [35].

Another outstanding question is what provides the friction that enables PGC motility. This is a complex question because of the different endodermal and mesodermal tissues PGCs migrate through. Either PGCs use specific adhesions and adapt them depending on the surrounding tissue, or they use non-specific adhesion, as commonly seen during amoeboid migration [21]. During migration through endodermal tissues, a plausible source of friction is via cell–cell adhesions (for a list of adhesion molecules expressed by PGCs in the mouse based on transcriptomic data, see [2]). Zebrafish PGCs generate friction at E-cadherin adhesions with surrounding cells to enable directional migration [25]. In the mouse, PGCs express E-cadherin during their specification and while they migrate in the mesoderm [36], as well as during later stages of migration when they traverse the mesentery [37] (Figure 2C). Based on immunofluorescence images, PGCs in the hindgut endoderm were proposed to lack E-cadherin [37]; however, this is impossible to assess when PGCs are within an E-cadherin expressing tissue. E-cadherin is present along the entire PGC membrane after they exit the hindgut endoderm at E9.5 and in protrusions into the mesentery made by PGCs still in the endoderm [37] (Figure 2C). PGCs in the endoderm may therefore indeed express E-cadherin (verifiable by transcriptomics or in situ hybridization), providing a possible source of friction for their movements in the endoderm. The surprising presence of E-cadherin at the PGC membrane in the E-cadherin-negative mesentery points to a possible role for this adhesion molecule in communication between PGCs [37]. Consistently, E-cadherin is enriched at contact sites between PGCs, and slices of E10.5 embryos cultured with an E-cadherin blocking antibody have fewer PGCs in the gonadal ridges and decreased PGC coalescence [37]. While these experiments do not reveal whether E-cadherin provides friction for migration, they raise interesting possibilities about PGC-PGC communication during migration.

### The ECM in PGC migration

During migration through mesodermal tissues, friction for migration may be generated through adhesions to the surrounding ECM. At E7.5, the mesoderm through which PGCs migrate contains fibronectin, laminin, and collagen IV [28,38,39] (Figure 2D). Recent work shows that these ECM components are enriched specifically in the region of the mesoderm that PGCs migrate through at E7.5 and that PGCs produce their own laminin throughout their migration [28]. The same components are found throughout the mesentery and around PGCs when they exit the hindgut [28,40] (Figure 2D). Based on culture assays, PGC adhesion to fibronectin decreases from E8.5 to E12.5, suggesting that fibronectin adhesion is important during migratory stages but not after [40,41]. Conversely, adhesiveness to laminin and collagen IV remains constant through these stages [40,41]. Proteoglycans are found at the basal surface of the hindgut endoderm and form a network of nodules in the dorsal mesentery along the PGC migratory path at E9.5 [42]. Proteoglycans are also detected on the surface of PGCs from E9.5 to E11.5 but not at the post-migratory stage of E12.5, suggesting that they may be important only during migration [42]. Overall, throughout their journey in mesodermal tissues, PGCs are surrounded by a complex ECM environment.

Whether the ECM around PGCs serves as a scaffold for migration or a barrier to it (or both, depending on context) is unclear. Fibronectin was detected by EM at contact sites between PGCs and somatic cells in the dorsal mesentery, suggesting that PGCs may use interstitial fibronectin as a substrate for adhesion-based migration [26]. Adhesion to the ECM is achieved through integrin binding, with the different integrin dimers binding to specific ECM components [43]. Recent work showed that the specification of PGCs at E6.5 involves detachment from basement membrane ECM and the absence of signaling via integrin β1 (*Itgb1*) [44]. Once PGCs are established, however, supplementation with these signals has no effect on PGC numbers, indicating that ECM signals are compatible with PGC maintenance [44]. Chimeric embryos containing *Itgb1^-/-^* PGCs show defective PGC migration, but only in the later stages of the journey [32] (Figure 2C). *Itgb1^-/-^* PGCs exit the endoderm but remain in the mesentery and fail to reach the gonadal ridges [32]. These data suggest that PGCs use integrin β1-dependent adhesion to the interstitial ECM to traverse the mesentery effectively. They also suggest that *Itgb1* is not required for migration of PGCs through the early embryonic mesoderm or the endoderm (Figure 2C). Whether other integrins are required for PGC migration at these earlier stages is unknown.

Single cell transcriptomics analysis of PGCs points to changes in the expression of genes related to cell-ECM adhesions from E9.5 to E10.5, as the overall population of PGCs shifts from predominantly migratory to relatively stationary within the gonadal ridges [45]. Comparisons between PGCs in different anatomical locations at E9.5 were also made based on the assumption that PGCs in the anterior represent the leading migrants (predominantly already in the mesentery) and those in the posterior represent lagging migrants (predominantly still in the hindgut) [45]. Genes involved in cell-ECM adhesion and actin polymerization were differentially expressed in anterior and posterior PGCs, reflecting possible differences in gene expression related to migratory state between leaders and laggers [45]. These differences may also reflect changes to the migration machinery or strategy as PGCs exit the hindgut. Further investigation of these transcriptional signatures is required to determine their functional relevance to PGC migration.

There is also evidence that the ECM around PGCs can hinder their migration. It is reasonable to assume that the more developed dorsal mesentery is more ECM-rich than the mesoderm of the early post-implantation embryo (Figure 2D), and live imaging studies indicate that PGCs move with an average velocity three times faster in the mesoderm at E7.5 than in the mesentery at E9.5 [9,11]. We can also draw on studies of mutant backgrounds with altered ECM deposition. In embryos lacking the downstream effectors of Bmp signaling, Msx1/2, PGC migration into the endoderm is impaired, with minimal dispersal of the PGC population along the hindgut and clusters of PGCs found towards the tail at E9.5 [46]. Msx1/2 mutants also have increased fibronectin in all tissues and in the basement membrane of the endoderm, which may impede PGC migration and cause them to be left behind in the posterior regions of the embryo [46]. Conversely, embryos lacking the TGFβ receptor Alk5 show a normal distribution of PGCs up to E9 but have increased numbers of PGCs exiting the hindgut and colonizing the gonadal ridges from E9.5 to E10.5 [47]. *Alk5^-/-^* mutants also have less collagen I around the hindgut, which may decrease the barriers to PGC migration and enable faster migration [47]. These studies suggest that interstitial ECM regulates the speed of PGC migration, with increased ECM leading to PGCs getting left behind as the embryo develops, and decreased ECM enabling them to reach their destination sooner.

The ECM also poses a potential barrier to PGC migration in a different form: as the basement membrane of the endoderm. As PGCs arrive at the endoderm, no significant basement membrane is detected by EM [18] or immunofluorescence [28], suggesting a lack of a structural barrier to PGC entry. However, by E9.5, the hindgut endoderm basement membrane has formed and includes collagens, fibronectin, and laminin [26,40,48] (Figure 2D). Since basement membranes typically keep tissues separate, the basement membrane of the hindgut endoderm probably acts as a barrier to PGC exit at E9.5. In line with this, PGC entry to the endoderm at E7.5, before a significant basement membrane has formed, is often transient and does not involve much PGC deformation—conversely, PGC exit at E9.5 is a one-way trip that involves much more dramatic deformation [28]. Basement membrane crossings occur in normal development, homeostasis, and cancer metastasis [49,50]. Depending on the context, these may involve protease-mediated degradation of the basement membrane and/or mechanical forces to push through it. PGCs express a variety of MMPs and related proteins [29], which could be used to degrade the basement membrane of the hindgut. Mechanical forces are equally plausible and may act alongside MMP activity to enable PGC exit from the endoderm.

PGCs extend long protrusions into the mesentery before they exit the hindgut endoderm [27,28]. These protrusions eventually widen, followed by the exit of the cell body from the endoderm and into the mesentery [28]. The function of these protrusions is unknown, however. They could be searching for chemotactic signals, probing their environment to find an exit route providing minimal resistance, or proteolytically or mechanically clearing a path for the cell body to follow. Given the importance of crossing the basement membrane to continue their journey, PGCs may have access to a variety of tools to accomplish this task, much like the anchor cell in *Caenorhabditis elegans* [51]. In mutants lacking MMPs, the anchor cell still breaches the basement membrane by upregulating actin polymerization and recruiting mitochondria to the invasive protrusion to power the growth of actin networks [51]. High-resolution live imaging and pharmacological inhibitors are required to determine the mechanism(s) by which PGCs cross the hindgut basement membrane.

### Morphogenesis along the PGC migratory path

Throughout PGC migration, the mouse embryo undergoes significant growth and morphogenesis, with multiple events occurring directly on the PGC migratory path. Some of these events, like the development of the hindgut basement membrane, may hinder PGC migration, while others may facilitate it. The clearest example of the latter is the formation of the hindgut, which sweeps PGCs within the posterior endoderm into the embryo. In Sox17 mutant embryos, the hindgut fails to invaginate, and PGCs remain at the embryo surface, demonstrating the importance of this morphogenetic event for PGC migration to the gonadal ridges [7]. Proper formation of the hindgut at later stages of development may be important as well. Embryos with a null mutation in Ror2, an important component of the planar cell polarity pathway, have fewer PGCs at the gonadal ridges at E9.5 [52], but whether this defect is cell intrinsic and/or due to overall defects in Ror2 null embryos is unclear. PGCs express Ror2 during migration, and Ror2 mutant PGCs are slightly less responsive to chemotactic gradients in culture and show impaired elongation and polarization [52]. However, Ror2 mutants also have altered hindgut morphogenesis, characterized by a much wider hindgut diameter [52], which increases the distance PGCs must migrate to reach the gonadal ridges. Ror2 phenotypes in PGC migration may therefore be due to both cell intrinsic effects and defects in the morphogenesis of the surrounding tissue.

While hindgut morphogenesis is required for the overall journey, we must also consider how changes in the architecture and mechanical properties of the endoderm influence PGC migration within it. Prior to hindgut formation, the endoderm is a thin layer of mostly cuboidal cells, but by E9.5, it begins to resemble an epithelium with more elongated and tightly packed cells [53] (Figure 2E). These morphological changes are accompanied by a decrease in intercellular space and an increase in stiffness measured by atomic force microscopy [28] (Figure 2E). PGCs migrate with similar average velocity in the hindgut endoderm as the mesentery, suggesting that their movements might be similarly hindered. Confinement and tissue stiffness are both important regulators of cell migration *in vivo* [54]. How the development of the hindgut endoderm into an epithelium influences PGC migration has yet to be explored. An intriguing possibility is that changes in tissue organization and mechanical properties of the hindgut serve as a signal for PGCs to make their exit at the correct developmental stage.

PGC exit from the hindgut and migration through the mesentery occurs over the course of morphogenesis of the dorsal mesentery, which gradually moves the hindgut further away from the dorsal wall [40] (Figure 1B). Development of this tissue involves high levels of cell proliferation [53], which may lead to increased cell density. Compared with the mesoderm at E7.5, the mesentery at E9.5 has smaller intercellular spaces and higher tissue stiffness, potentially hindering PGC migration [28] (Figure 2E). Morphogenesis of the mesentery also leads to increased migration distance for PGCs that leave the hindgut later (Figure 1B). This final leg of the PGC journey, which already appears to pose obstacles and slow them down, also becomes even longer and more difficult over developmental time.

### Confinement and the nucleus in PGC migration

The changing environment of the developing embryo may impose increasing confinement on PGCs over the course of their migration. In such confined environments, the nucleus can either hinder or propel cell migration [55]. A large and stiff nucleus may be difficult to squeeze through tight spaces, causing its movement to be a rate-limiting step for cell migration [55]. In other contexts, it can facilitate migration into confined spaces, such as in leukocytes crossing endothelial cell layers [56] or border cells migrating between tightly packed nurse cells in the *Drosophila* egg chamber [57]. The former is associated with nuclear positioning at the rear of the cell, while the latter is associated with the nucleus at the front [55]. The role of the nucleus in PGC migration is unknown, but combining observations of PGCs with newer findings in other systems can lead to the generation of interesting hypotheses. Classic EM studies show that PGCs migrating through the mesentery tend to have their nucleus positioned at the rear of the cell [18], consistent with migration hindered by the nucleus. Live imaging shows that PGC nuclei undergo dramatic but transient deformation during migration, with the nucleus trailing behind the protrusion at the cell front [28]. How do PGCs, and specifically their nuclei, adapt to the increasingly confined environment of the developing embryo? In culture models, cancer cells migrating through narrow microfluidic channels experience extreme deformation of the nucleus that can lead to nuclear rupture and subsequent DNA damage [58]. Further, mechanical compression alone can induce DNA damage via replication stress without any nuclear rupture [59]. During their long migration through the hindgut endoderm and mesentery, it seems that PGCs encounter tight spaces they must squeeze through, posing a risk to their nuclear integrity.

In rare cases, PGCs have been seen to suddenly rupture following migration into or through the mesentery [28]. These events occur only in later stages of migration when the somatic tissues surrounding PGCs are more developed [28]. These later stages also coincide with increased incidence of DNA damage specifically in PGCs—not in the less migratory somatic cells around them [28] (Figure 2F). Importantly, the frequency of rupture events and DNA damage in PGCs increases when confinement is pharmacologically increased through inhibition of MMP activity [28]. These observations raise important questions about genome integrity and DNA damage repair during PGC development. In embryos lacking one of several different DNA damage repair components, including *Ercc1*, *Fanca*, and *Rev1*, development proceeds normally in all somatic cells, but PGC numbers are lower at E12.5 and as early as E9.5 in the case of *Rev1* [60,61]. This phenotype is caused by the accumulation of DNA damage specifically in PGCs, which are then eliminated by apoptosis [60]. These findings raise the question of how DNA damage arises in PGCs at these early, pre-meiotic stages of their development. One possibility is via metabolically produced aldehydes; *Fanca^-/-^* mutants lacking genes involved in aldehyde detoxification have even fewer PGCs at E12.5 and increased DNA damage in PGCs [60]. Another is that the increasing confinement imposed by the developing embryo leads to DNA damage due to nuclear rupture and/or mechanically induced replication stress, as seen in culture [58,59]. Such a phenomenon is rarely seen in embryos, making this an exciting area to explore.

If the journey undertaken by PGCs poses risks to their nuclear integrity, it is possible that strategies to mitigate this risk have evolved. For example, PGCs may adapt their nuclei to facilitate confined migration and prevent damage. The mechanical properties of the nucleus, such as the composition of the nuclear lamina, influence confined migration in culture [62]. Early EM studies show that PGCs possess irregularly shaped nuclei, particularly by E9.5 [18], possibly indicative of a softer nucleus which would ease migration under confinement. Recent work shows that PGCs lack Lamin A/C and gradually deplete Lamin B1 over the course of migration (Figure 2F), and this remodelling of the nuclear lamina could explain the highly wrinkled envelopes of PGC nuclei [28]. Changes to chromatin organization can also affect nuclear mechanics [63]. Migrating PGCs undergo extensive epigenetic reprogramming and chromatin remodeling, which are thought to help maintain their latent pluripotency [64,65]. As PGCs arrive at the endoderm (E7.5-E8), H3K9me2 methylation decreases, and as PGCs exit the hindgut at E9.5, H3K27me3 methylation increases [65]. These changes in methylation are associated with differences in repression and chromatin organization: H3K9me2 reflects broad repression and is associated with heterochromatin, while H3K27me3 is associated with plastic repression and euchromatin [65]. Epigenetic remodeling therefore has important implications not just for transcription in PGCs but also perhaps for the mechanical properties of the nucleus: for example, increased euchromatin (and decreased heterochromatin) softens the nucleus of mouse embryonic fibroblasts [63]. Changes in DNA and histone methylation in PGCs could lead to softening of the nucleus and facilitate their migration through developing tissues. Overall, careful mechanical measurements of the nucleus and perturbations to the lamina and to chromatin are needed to determine the physical properties of the PGC nucleus and the factors underlying them.

The signals that regulate the timing of these epigenetic remodeling events are not fully known, but their progression correlates with PGC migration through increasingly developed tissues. It is possible that physical signals are integrated with molecular ones to orchestrate chromatin reorganization at the right time and place. Mechanical stresses can induce changes in methylation and chromatin organization in cultured cell layers and in intact tissues [66]. Stretching of cell layers leads to a decrease in H3K9me3 methylation and heterochromatin and a gradual increase in H3K27me3 methylation, which softens the nucleus and protects the cell from DNA damage [66]. Decreased H3K9me3 methylation is also associated with nuclear wrinkling [66], similar to PGCs at E9.5 that have low levels of H3K9me3 methylation [64] and irregularly shaped nuclei [18]. How migration through developing tissues affects the nucleus of PGCs is unknown, but it is an important line of inquiry. As these essential cells will be the only ones to contribute to the next generation, the preservation of their nuclear and genome integrity during their long journey is of vital importance.

## Conclusion and outlook

There are myriad unanswered questions about how PGCs migrate through the developing mouse embryo, but with modern techniques, it is time to delve into this remarkable journey. Apart from advances in mouse models and imaging, molecular biology and omics techniques are becoming more powerful and adept at handling rare cell populations. PGCs in the mouse embryo provide an exciting model of mammalian cell migration *in vivo*, given the length of their journey and the diverse environments they encounter. PGC migration is also an important system to understand more fully, as we do not know how migration affects individual PGCs and their subsequent development, or the final makeup of the germline as PGCs are lost or damaged along the way.

**Perspectives**

- The migration of primordial germ cells (PGCs), which eventually give rise to sperm and egg, is essential for fertility and evolutionarily conserved, albeit with differences in routes and migration strategies in different species. Little is known about this process in the developing mammalian embryo, including the cellular mechanisms of migration and the impact that migration has on germ cell development.
- PGCs in the mouse migrate using long protrusions and are surrounded by the ECM at different stages of their journey, possibly using a mode of migration distinct from that used in better-studied model organisms. Their journey is influenced by the morphogenesis of tissues they migrate through, in some cases aiding and in others hindering their progress.
- With advances in imaging and the growing repertoire of fluorescent reporter mice, it is now possible to delve into this understudied process. Future work should determine the cellular mechanisms of PGC migration, if and how PGCs adapt their migration mode to different tissue environments, and how the PGC nucleus behaves during migration to preserve genome integrity.

## Abbreviations

E: embryonic day
ECM: extracellular matrix
EM: electron microscopy
MMP: matrix metalloproteinase
PGC: primordial germ cell
